# Increased homozygosity in the first Hispanic patient with plantar lipomatosis, unusual facies, and developmental delay (Pierpont syndrome): a case report

**DOI:** 10.1186/s13256-016-0997-1

**Published:** 2016-08-12

**Authors:** Siobhán O’Keefe, Dieter T. Wefuan, Jennifer B. Humberson, Karen Schmidt, John Wiley

**Affiliations:** 1 Department of Pediatrics, Brody School of Medicine, East Carolina University, 3E-139 Brody Medical Sciences Building, Greenville, NC 27834 USA; 2 Vidant Duplin Hospital, 401 N Main St, Suite C-9, Kenansville, NC 28349 USA; 3 Department of Pediatrics, University of Virginia, Charlottesville, VA USA

**Keywords:** Case report, Pierpont syndrome, Plantar lipomatosis, Prominent digit pads, Developmental delay, Widely spaced teeth, Large ears, Excess plantar skin, Supernumerary nipples, Increased homozygosity

## Abstract

**Background:**

Pierpont syndrome was first described in 1998 with key characteristics including developmental delay, dysmorphic facial features, fat pads on hands and feet, and feeding difficulties. To date the mechanism of inheritance is unknown. Nine out of ten previously described patients with Pierpont syndrome were boys. This is the first report of a case of a non-white patient with Pierpont syndrome and she is the second female patient to be described as having Pierpont syndrome.

**Case presentation:**

Our patient is a 16-month-old Hispanic girl with extreme developmental delay, microcephaly, large ears, short and thick upper lip, broad philtrum, widely spaced teeth, constipation, dysphagia, fat pads on feet and hands, autistic behavior and seizure-like episodes. She had a normal karyotype (46,XX), and array testing showed greater than 8 % homozygosity with otherwise normal results. Genes within these areas of homozygosity may provide clues to an etiology and suggest autosomal recessive inheritance. This case report highlights the possibility of ethnic variations in this syndrome’s presentation, which may have ramifications in uncovering the pathogenesis as well as expanding the phenotype.

**Conclusion:**

Pierpont syndrome should be considered in the evaluation of children with the described features, regardless of their gender and ethnicity.

## Background

The combined characteristics of plantar lipomatosis, unusual facies, and developmental delay are known as Pierpont syndrome [PS]. There have been a total of ten cases of PS reported since its initial description [1–3]. Key characteristics of the syndrome include neurodevelopmental delay, dysmorphic facial features, fat pads on feet and hands, and feeding difficulties. The mechanism of inheritance is unknown. All previous cases described have been in the white race of which nine out of ten were boys. Our patient represents the first non-white and the second female to be identified with PS.

## Case presentation

A 16-month-old Hispanic girl presented to a genetics clinic with developmental delay and abnormal facial features. She was born at term to unrelated parents. Her mother and father’s ages were 29 and 30 years respectively. Her two older sisters are both healthy with normal development. There is no family history of a child with intellectual disability or similar findings. The maternal grandmother had a baby who died at 1 week of age with multiple birth defects.

Developmental delay was first noticed at 4 months of age and she was referred for early interventional therapy. When she was first seen in the genetics clinic at 16 months, she only made whining noises, did not make eye contact, and could not sit upright, crawl, or rollover. She made poor eye contact and had episodes of inconsolable agitation with self-mutilation. She had constipation, abdominal bloating, and only tolerated pureed food. She developed a periorbital urticarial rash and tested positive for multiple food allergies.

**Fig. 1:**
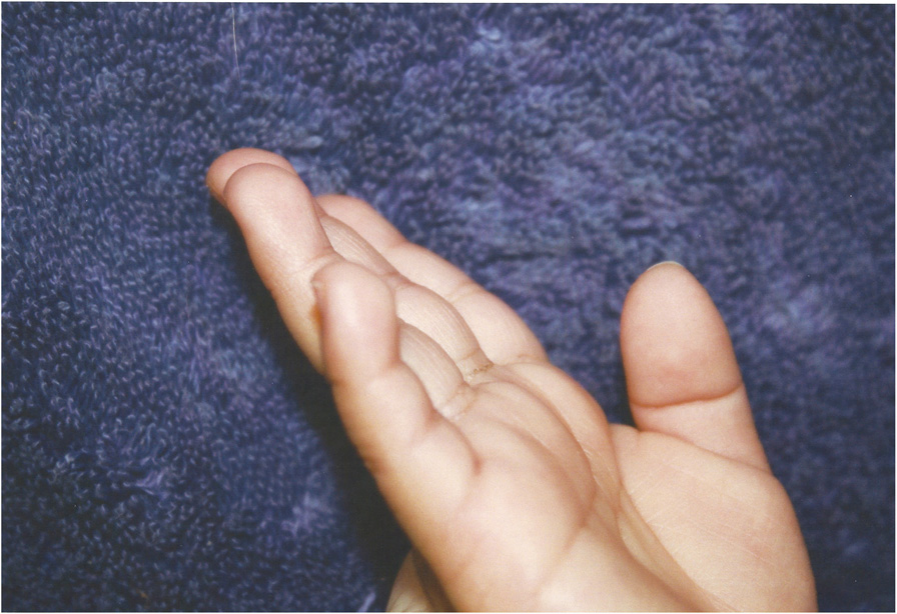
Fetal finger pads and pillowing of palm between palmar creases

**Fig. 2:**
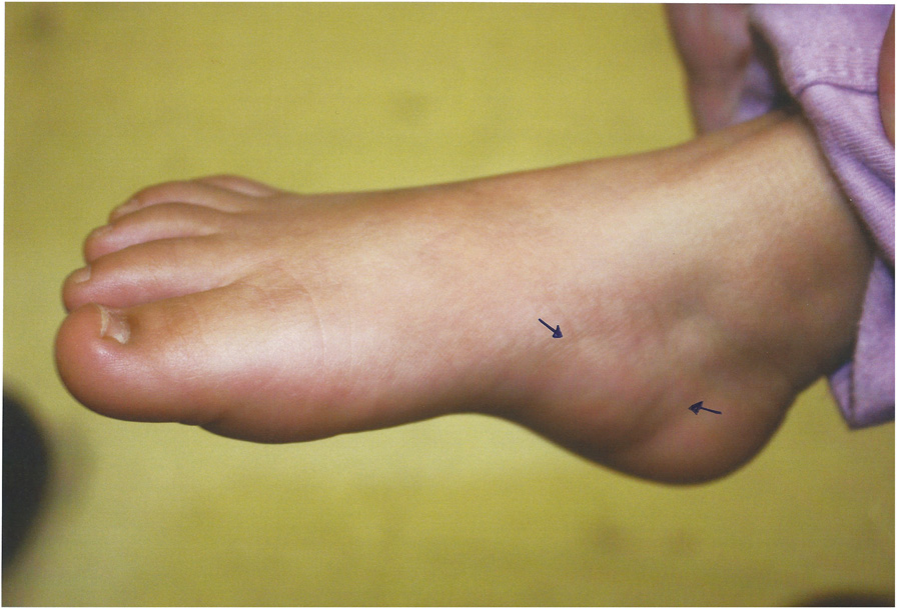
Anteromedial fat pad (between arrows)

Her head circumference was at the fifth percentile compared to her length at the 25th percentile. Her ears were large at the 95th percentile. She had two skin papules directly above her nipples bilaterally (supernumerary nipples) and one lateral to her right palpebral fissure. She had mild midface hypoplasia, anteverted nares, short thick upper lip, broad philtrum, protuberant lower lip, high palate, and widely spaced teeth. She had an abnormal hair whorl offset to the side. She had prominent digit pads on her middle three fingers bilaterally (Fig. 1). She had excess skin on the plantar surface of her feet, sole creases were few in number, and there was a palpable soft fat pad anteromedial to the heel of both her feet (Fig. 2). Dr Pierpont reviewed her photos and confirmed that her features were compatible with PS.

She had a normal karyotype (46,XX). A chromosomal microarray analysis (CMA) using the Affymetrix Genome-wide Human Single Nucleotide Polymorphism (SNP) Array 6.0 revealed 118 independent regions of homozygosity. These ranged from 1 Mb to 5.8 Mb and encompassed 8 % of her genome. She was enrolled in a whole exome study run by Baylor College of Medicine, USA.

## Discussion

Features in our patient that are similar to other children with PS include developmental delay, midface hypoplasia, large ears, widely spaced teeth, prominent digit pads, fat pads anteromedial to the heels, and feeding difficulties [1–4]. Features that are unique to our patient include neutral palpebral fissures, normal nose size, and supernumerary nipples. These differences may represent ethnic or gender variations that allow for the expansion of the PS phenotype.

All previously described patients with PS had normal karyotypes. The patient described by Oudesluijs *et al*. in 2005 had a normal whole genome microarray [1]. Of the seven patients described by Wright *et al*. in 2011, three did not have array investigations, three had normal array studies, and one had a 250K array with a 3.17 Mb loss at 10q21.3 that was verified by fluorescence *in situ* hybridization (FISH) in a monozygotic twin and their mother [4].

The numerous areas of homozygosity seen in our patient indicate that autosomal recessive genes may have a role in PS. We identified 172 recessive genes in the homozygous areas using an SNP array evaluation tool [5]. A comparison of clinical findings in our patient and those associated with the recessive genes in the areas of homozygosity revealed four recessive genes with overlapping findings. These genes are listed in Table 1. The major phenotypic findings shared with PS include developmental delay, feeding difficulties, and dysmorphic facial features. Lipomatosis was not a feature of any phenotypes associated with recessive genes in the area of homozygosity.

**Table 1:** Four of the 172 recessive genes in the areas of homozygosity are associated with phenotypes similar to our patient

Gene	Associated syndrome or phenotype gene
*ARID1A* gene at 1p35.3	Coffin–Siris syndrome
*PIGV* at 1p36.11	Hyperphosphatasia with mental retardation
*POC1A* at 3p21.2	Short stature, onychodysplasia, facial dysmorphism, and hypotrichosis (SOFT) syndrome
*CDK5RAP2* at 9q33.2	Microcephaly 3

## Conclusions

The mechanism of inheritance of PS is unknown. The finding of 8 % homozygosity in this patient may suggest autosomal recessive inheritance. PS should be considered in children with the described features, regardless of their gender and ethnicity.

## Abbreviations

CMA, chromosomal microarray analysis; FISH, fluorescence *in situ* hybridization; PS, Pierpont syndrome; SNP, single nucleotide polymorphism; SOFT, short stature, onychodysplasia, facial dysmorphism, and hypotrichosis
